# Digital support for female students in physical education universities in Japan

**DOI:** 10.1038/s41598-025-98921-0

**Published:** 2025-05-14

**Authors:** Junko Okuyama, Shuji Seto, Tomonori Motokawa, Tomomi Kato, Aya Miyamoto, Maki Maekawa, Shunichi Funakoshi, Tatsuma Okazaki, Satoru Ebihara

**Affiliations:** 1 Department of Human Health and Nutrition, Shokei Gakuin University, 4-10-1 Yurigaoka, Natori, Miyagi 981-1295 Japan; 2 Faculty of Informatics and Data Science, Akita University, 1-1 Tegatagakuen-machi, Akita-shi, Akita Japan; 3 Frontier Research Center, POLA Chemical Industries, Inc., 560 Kashio-cho, Totsuka-ku, Yokohama, Kanagawa Japan; 4 Department of Athletic and Sport Sciences, International Pacific University, 721 Kannonji, Seto-cho, Higashi-ku, Okayama City, Okayama Japan; 5 Nara National Institute of Higher Education and Research, Nara University of Education, Takabatake-cho, Nara City, Nara Japan; 6 Arimakougen Hospital, 4663-3, Kamitsu, Nagao-cho, Kita-ku,, Kobe,, Hyogo Japan; 7 Department of Rehabilitation Medicine, Tohoku University Graduate School of Medicine, 1-1 Seiryo-machi, Aoba-ku, Sendai City, Miyagi Japan

**Keywords:** Mood stabilization, Female athletes, Psychological state, Facial data, Premenstrual syndrome, Insomnia, Psychology, Health care, Engineering

## Abstract

Alongside acquiring specialized knowledge and accomplishing developmental tasks, athletic colleges require young athletes to also be active. We investigated the use of a smartphone application, ME-FULLNESS, as an unprecedented support method for female college students currently enrolled in athletic colleges. ME-FULLNESS is an application that infers one’s psychological state from their facial information and improves their psychological state with music, vibration, and images that match that psychological state. We conducted a psychological survey with purposively selected female university students (18 to 24 years) at the International Pacific University in Okayama, Japan, before and after one month of using ME-FULLNESS (*N* = 76) and a group of non-users (*N* = 25). The app-using group showed significant improvement in depressive symptoms (*p* = 0.002), anxiety symptoms (*p* = 0.000), stress (*p* = 0.000), insomnia (*p* = 0.002), severity of premenstrual syndrome (*p* = 0.000), and resilience scores (*p* = 0.000), while the non-app-using group showed improvement in anxiety (*p* = 0.009) and resilience scores (*p* = 0.000). This study suggests that using the ME-FULLNESS app may improve depression, stress, insomnia, and resilience among athletic female students, positively contributing to their college life and sports performance.

## Introduction

University and college students experience a critical developmental period characterized by sudden freedom and challenges^1^. They face two major challenges: The first involves acquiring professional knowledge to prepare for a healthy and high-quality future career^2^ and the second involves developing in a manner that is appropriate for young adulthood^3^. University and college students exhibit developmental changes in self-esteem^4^, which are important for interpersonal development^5^.

University or college student athletes are required to continue competing while dealing with various issues, such as those stated above, while also balancing schoolwork and post-graduation career paths; when these issues are not addressed successfully, they frequently develop various psychological problems that interfere with their athletic life^6^. Elite female athletes are expected to perform at their peak during competitions; any negative effects of female-specific issues or menstrual cycle-related inconveniences could affect their performance^7^. Female athletes experience problems with a triad of interrelated signs, including inadequate energy supply, with or without eating disorders; decreased bone density; and menstrual dysfunction^8,9^. Other common sex-specific issues that affect athletes include breast injuries, pelvic floor issues, saddle sores, and menstrual symptoms^10^.

Previous research on improving athletic performance and recovery in athletes aged 18 to 24 years has shown that extended sleep has the most beneficial effect on subsequent performance^11^. For this reason, it is necessary to establish guidelines and intervention tools to help athletes deal with their specific sleep requirements and sleep disorders. There are also many intervention strategies that focus mainly on personalized programs including psychological skills training, coping and optimism training, mindfulness, yoga, general relaxation, imagery, and combinations of both, for the mental toughness that affects an athlete’s performance^12,13^. However, because of the difficulty in evaluating the effectiveness of interventions targeting athletes, there are no established intervention methods yet.

**Fig. 1:**
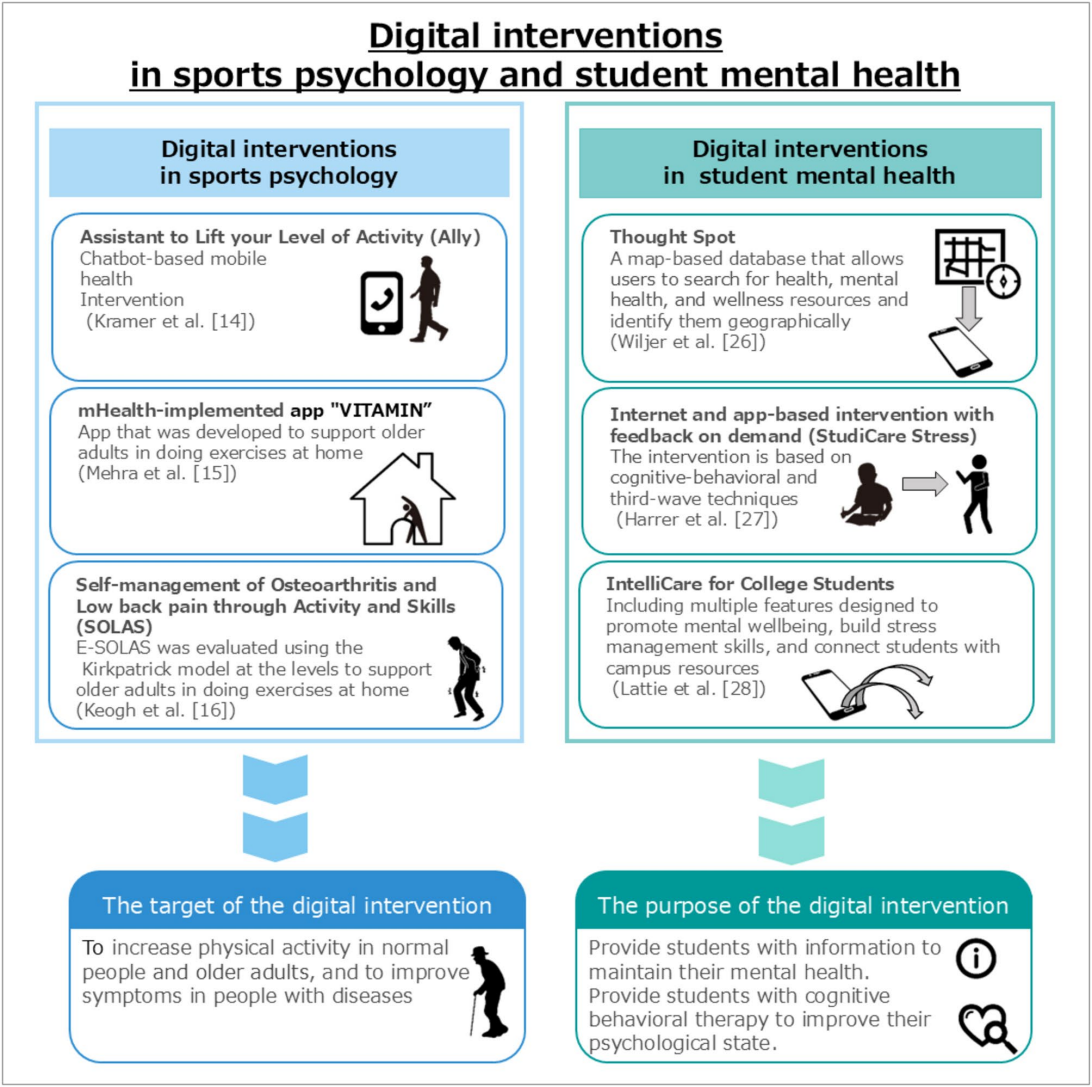
Digital interventions in sports psychology and student mental health.

Therefore, it is important to utilize current technology and knowledge to support female athletes in physical education universities or colleges. Current examples of digital interventions in sports psychology include the Assistant to Lift your Level of Activity (Ally) app^14^, the mHealth-implemented app “VITAMIN”^15^, and the Self-management of Osteoarthritis and Low back pain through Activity and Skills (SOLAS)^16^, as shown in (Fig. 1). The Ally app is a smartphone application that combines financial incentives with chatbot-guided interventions to encourage users to reach personalized daily step goals^17^. There is also the app Carrot Rewards that adds a function to reward apps that encourage individuals to achieve physical activity, such as Ally^18^ and Google Fit^19^. The tablet computer application “VITAMIN” was developed to help older people follow a personal training program^15^. Older adults, especially those aged 50 to 99 years, women, and those who are wealthy (high income, high education, digital skills) are reported to be using or planning to use digital technology independently at home or in the community for an average of three months to improve their health and prevent disease^20^. SOLAS, underpinned by the self-determination theory, was designed to support an increase in self-management behavior in patients with chronic low back pain (CLBP) and osteoarthritis (OA) in primary care physiotherapy^16^. Other digital interventions that support patient self-management in primary care include “HOME BP,” an intervention for hypertension, and “My Breathing Matters,” an intervention for asthma^21^. These digital interventions have mainly aimed to increase physical activity among older adults and those with illnesses, and do not intend to further improve the performance of young athletes.

Examples of digital interventions for university students are also shown in (Fig. 1). Among university students, 25–50% meet the criteria for at least one mental disorder in a year^22,23^; mental health measures are important in this age group as mental disorders are associated with various negative effects, such as a decline in academic performance^24^ and university dropout^25^. Mobile health technology is being touted as a promising solution to address students’ unmet mental health needs and includes the Mobile and Web App “Thought Spot”^26^, an internet and app-based intervention with feedback on demand, “StudiCare Stress”^27^, and a self-guided app-based mental health platform “IntelliCare for College Students”^28^. In Japan, several digital tools have been developed for the university student generation, such as the student support chatbot called “Dabot: Mr. Dax’s Counseling Room”^29^. Tools like Thought Spot^30^, IntelliCare for College Students^28^, and Dabot help students seek support from mental health and wellness resources. Digital interventions allow users to find and share resources through a database on the device and provide a private space for users to track their thoughts and moods. In addition to the resource navigation support, there is also HEARTSMAP-U, which allows individuals to self-assess psychosocial issues across 10 Sect^31^. The StudiCare Stress intervention is based on cognitive behavioral therapy and third wave therapy and is consistent with Lazarus’s transactional model of stress. The StudiCare Stress intervention comprises eight main modules. Completing one module takes 30 to 90 min, and participants are advised to complete at least one and a maximum of two modules per week. As a similar product to StudiCare Stress, an internet-based, guided online self-help program for distressed university students is based on standard cognitive behavioral therapy principles and consists of five core modules, some of which include options that focus on anxiety, depression, and stress^32^. University students who play sports have little free time due to their studies and sporting activities; thus, they do not have the time to search for ways to solve their problems through apps or to do their homework using cognitive behavioral therapy. Furthermore, female athletes face difficulty resolving the areas and related health issues that may affect their participation in sports, performance, and health outcomes^33^.

**Fig. 2:**
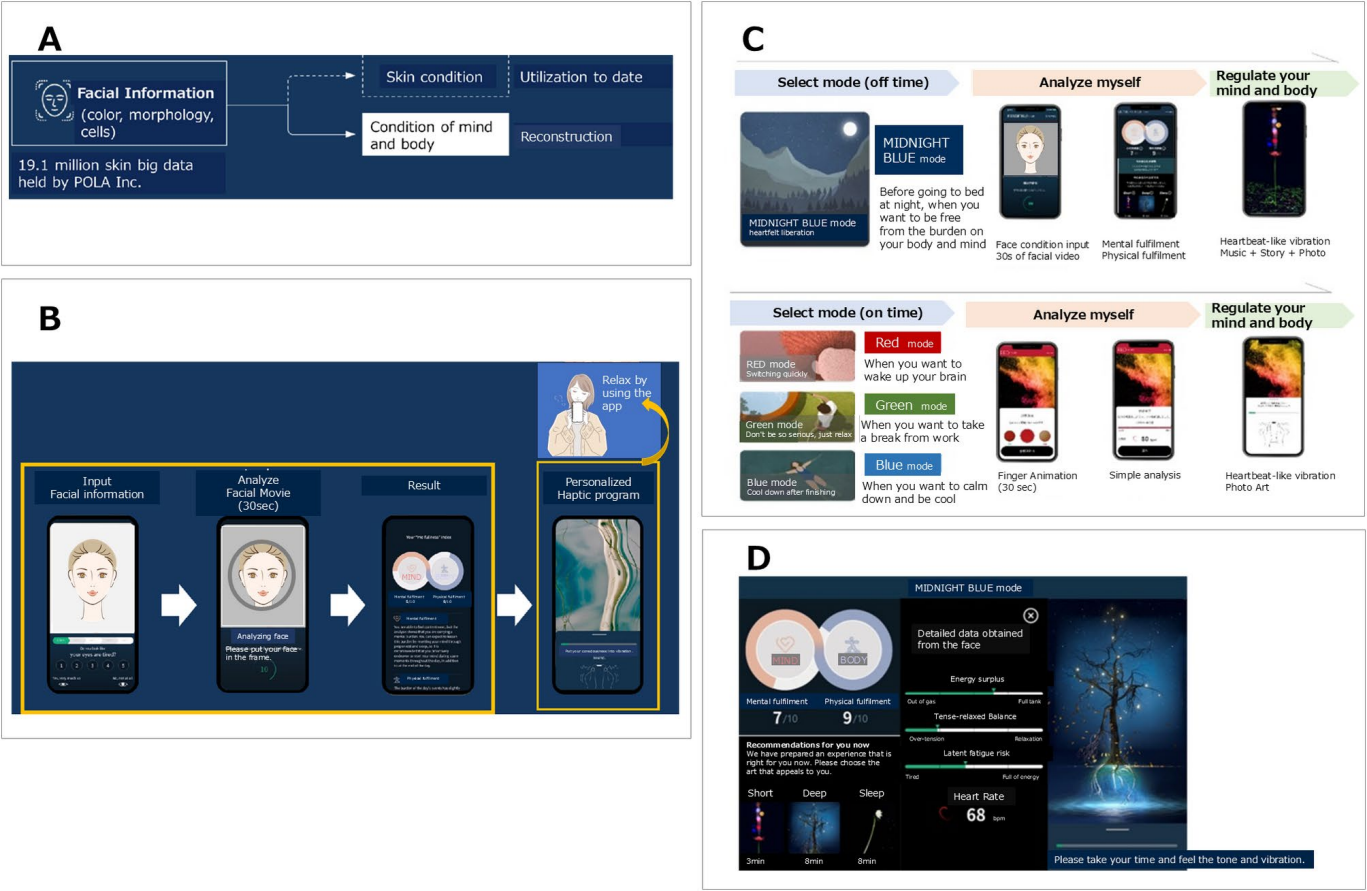
ME-FULLNESS application. (**A**) Estimation of psychological state based on facial information; (**B**) Flow of ME-FULLNESS app; (**C**) Off-time and on-time modes in the ME-FULLNESS app; (**D**) Details of the ME-FULLNESS app’s off-time mode.

The ME-FULLNESS app has been proven to be effective in reducing symptoms of depression, stress, and anxiety in the general adult population^34^. ME-FULLNESS is a quick and easy-to-use app that provides management strategies (e.g., relaxation, mood activation) based on mood estimated from facial information. In the “On time” mode, which is used during the day, the smartphone vibrates in time with the heart rate estimated from the blood flow in the finger, and the gradual increase in the speed of the vibration has a mood-lifting effect. In the “Off time” mode, which is used before going to bed, a program that changes mood is provided in line with the physical and mental state estimated from the information regarding the user’s face, such as blood flow in the cheeks, facial color, shape of facial cells, and water content, using a smartphone to estimate the user’s psychological state (Fig. 2A); the user can experience mood changes to reduce symptoms of depression and anxiety through music or readings that can be played on the smartphone (Fig. 2B). Although cognitive behavioral therapy is effective in engaging users in mobile health interventions, it is time-consuming and labor-intensive, and students at a physical education university have very limited time to devote to improving their mood. Fully automated interventions are a non-depleting resource in that they can benefit busy students without requiring time or effort. For this reason, we thought that if students at a physical education university used ME-FULLNESS, an intervention that could be used independently by students without consuming a lot of time or effort, it would improve their psychological state to the maximum extent. Moreover, it is important for university students to take medication, seek social support, or engage in activities that induce positive emotions as coping strategies for controlling premenstrual syndrome (PMS). We thought that adapting ME-FULLNESS to female university students at a physical education university and inducing positive emotions might improve PMS.

We selected the International Pacific University (IPU) as our research target university because IPU is the only university in Chugoku and Shikoku in Japan that has a physical education department and encourages students to engage in sports activities based on the educational philosophy of integrating education and sports. Numerous current students belong to physical education clubs and participate in athletic activities daily. Some have even won national or world championships^35^. We believe that female students, who are expected to perform at a high level in sports and academics at university, require increased psychological support.

To maintain sports performance and acquire and develop college knowledge, psychological support must be provided through modern science. Therefore, this study’s objectives are as follows: (a) exploring how to facilitate mood stabilization among current students in athletic colleges and (b) examining the effectiveness of a smartphone application for mood stabilization among female athletes.

## Methodology

This comparative study evaluated the ME-FULLNESS app’s effectiveness for female students at a physical education college. The effect of successive, chronological interventions was estimated in this study using a non-randomized, before-and-after intervention study. This study was conducted between November 9 and December 20, 2023. The participants were informed regarding the study’s purpose and methods in advance; written informed consent was obtained from all participants. This study was approved by the Ethics Committee of Tohoku University Graduate School of Medicine (Approval No. 2023 1 544) and the study protocol was pre-registered with the University Hospital Medical Information Network Center (UMIN study ID: UMIN000052125; First registration date: September 11, 2023). All study procedures were conducted per relevant guidelines and regulations.

### Participants

The sample size was estimated based on similar studies by Schichido^36^ and Lui et al.^37^ on the effect of interventions on outcomes. Therefore, it was necessary to recruit at least 64 participants in the group using ME-FULLNESS.

**Fig. 3:**
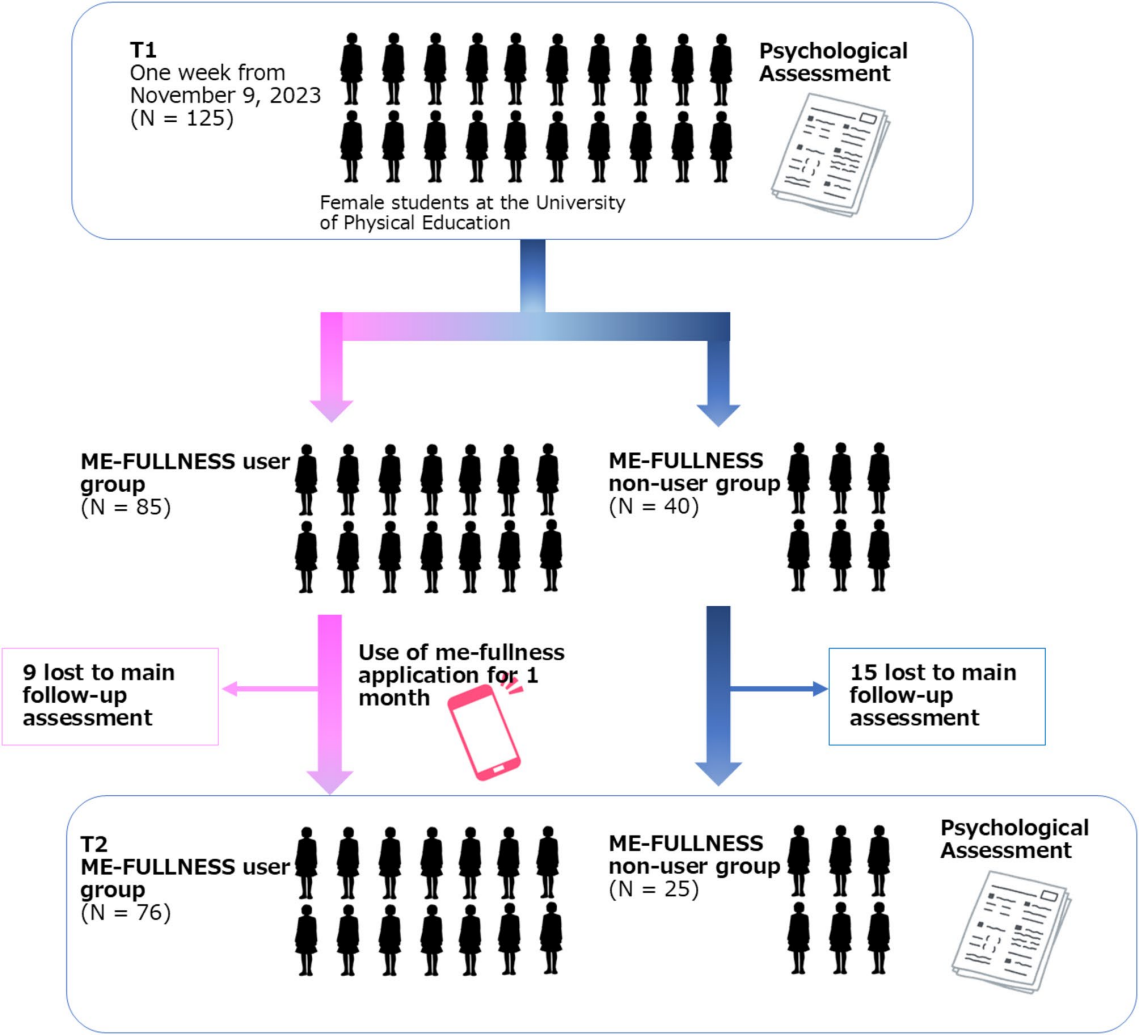
This study’s flow.

Female students aged 18 to 24, from the University of Physical Education, IPU, Okayama, Japan, were selected. A poster outlining the study was presented, and 125 people were invited to participate. They were given a summary of the study’s aim and procedure and screened for eligibility as per the inclusion/exclusion criteria below. Table 1 presents the study participants’ background characteristics. Of these, 85 and 40 were assigned to the application (ME-FULLNESS) use group and non-use group, respectively, for one month. At the one-month follow-up assessment, the number of participants in the ME-FULLNESS app use group had increased to 76, while that in the non-use group had decreased to 25. Figure 3 illustrates this study’s flow.

**Table 1:**
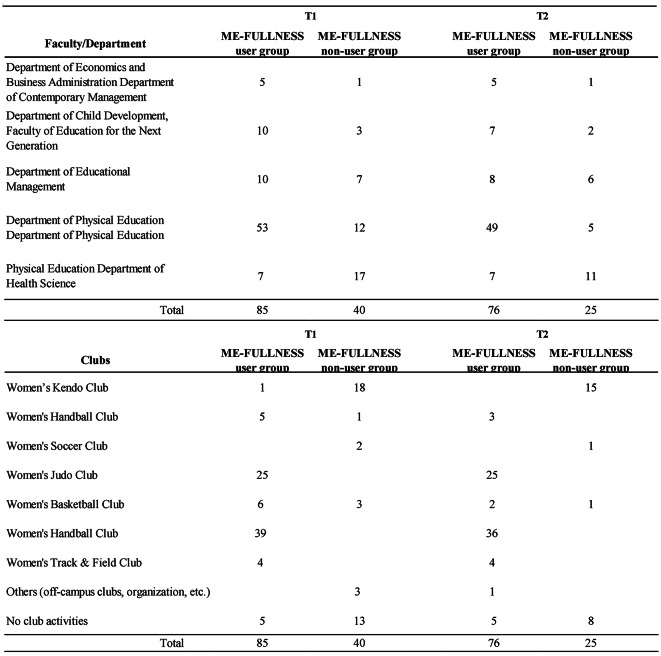
Details of the research participants.

#### Inclusion criteria

A. Female university students at the IPU, aged between 18 and 24 years.
B. Must indicate their intention to participate in the study and agree to participate in the study after understanding the explanation.
C. For the ME-FULLNESS app group, must own an iPhone with sufficient free space (approximately 800 MB or more) to install the app (iOS13 or higher, iPhone8 or higher).

#### Exclusion criteria

A. Taking medication daily that may affect the evaluation of this test (medication that claims to improve fatigue, stress, or sleep).
B. Having a hearing problem that interferes with daily life.
C. Participating in other intervention studies.
D. Deemed inappropriate by the test supervisor.

### Tools

#### ME-FULLNESS app

The ME-FULLNESS app is a smartphone app that aims to utilize the skin analysis technology accumulated by POLA Chemical Industries, Inc. for the wellbeing of the mind and body (Fig. 2A). The app has a night mode for off-time and a day mode for on-time. The night mode is designed to soothe the mind and provide peaceful sleep, while the day mode is designed to support a change of mood and promote positivity. Both modes work by first analyzing the physical and mental state, and then using methods tailored to that state to help the individual relax and prepare for the next situation (Fig. 2C).

In the “off-time” mode, the user must first use the app to film a 30-second video of their face to assess their current state. The color of the user’s face changes every second due to the flow of blood, and by analyzing the state of this blood flow, the app can analyze the user’s heart rate and autonomic nervous system. It can also deduce the user’s current level of fatigue from the texture of their cheeks, any dullness or blemishes, and so on, and use this to evaluate their state of mind and body (Fig. 2D). Subsequently, three recommended contents are presented to soothe the senses with music and beautiful images, in line with the derived data. There are currently about 500 types of content, and they are set up so that the user can choose the one that best suits the time required and the state that the user wants to achieve. The device also vibrates per the heart rate at the time and the mode of selection. This vibration is like a gentle patting motion that the user may have experienced as a child before falling asleep, and all the music has been specially created to match the vibration. This gradually helps restore the user’s resilience to stress, allowing them to relax deeply (Fig. 2D).

Meanwhile, in the daytime “on-time” mode, the state analysis is set up so that it can be easily carried out by taking a video of the user’s thumb rather than their face. There are three modes available: Red Mode for those who want to clear their minds and revitalize themselves, Green Mode for those who want to relax in between work, and Blue Mode for those who want to soothe their irritability and return to a calm state (Fig. 2C).

#### Psychological test questionnaires

The items were selected from various validated psychological test questionnaires that investigate psychological states. These were all self-report questionnaires. One of the tools selected was the Depression Anxiety Stress Scale (DASS)-21, which was chosen because it is a single psychological test questionnaire that can consider depressive symptoms, anxiety symptoms, and stress. The state of insomnia was evaluated using the Japanese version of the athens insomnia scale (AIS), which is a valid tool based on the criteria of the 10th edition of the international classification of diseases.

PMS is a group of psychological and physical symptoms that appear during the luteal phase and resolve with the onset of menstruation. Around 30% of women of reproductive age are estimated to suffer from moderate to severe PMS^38^. Of the available assessment tools, the daily record of severity of problems (DRSP) is the most widely used and is also recommended as an effective measure in the guidelines of the royal college of obstetricians and gynaecologists (RCOG)^39^.

Resilience is a dynamic and multidimensional concept that refers to the ability of an individual to cope successfully and recover from adversity; it is associated with growth and overcoming challenges^40^ and means that individuals adapt positively despite past and everyday difficulties^41,42^. Physical education students may experience academic difficulties, frustration, and a desire to improve their performance in their chosen sport, alongside a lack of progress in their sporting achievements, which can result in a decrease in self-esteem and loss of confidence. Considering this situation, physical education students need to have sufficient resilience to cope with adversity and problematic situations^43^. We believe that the 10-item connor-davidson resilience scale (CD-RISC 10) is a useful assessment tool because it is one of the most widely used reliable and valid scale and allows for the quick and easy assessment of resilience.

##### Depressive, anxiety, and stress symptoms

The DASS-21 comprises 21 items, with seven items each measuring depression, anxiety, and stress^44^. Responses are scored as 0 (did not apply to me at all), 1 (applied to me to some degree or some of the time), 2 (applied to me to a considerable degree or a good part of the time), or 3 (applied to me very much or most of the time). Participants respond by selecting the alternative closest to their condition in the last week. Responses to each question (0–3 points) are tabulated; the scores for depression, anxiety, and stress for each factor item are calculated and evaluated. Multiple studies have used the DASS-21 to assess psychological outcomes^45^. We used the Japanese version of the DASS-21 (short-form version of the DASS-21) to assess participants’ levels of depression, anxiety, and stress^44,46^. Especially for the English version, the reliability (internal consistency) of the DASS-21 Anxiety, Depression, Stress, and Total scales was estimated using Cronbach’s alpha, which was 0.88 (95% CI 0.87–0.89) for the Depression scale, 0.82 (95% CI 0.80–0.83) for the Anxiety scale, 0.90 (95% CI 0.89–0.91) for the Stress scale, and 0.93 (95% CI 0.93–0.94) for the Total scale^45–47^. However, the reliability and validity of the Japanese translation of the DASS^44^ have not yet been reported^48^.

##### Sleep quality

The AIS is a straightforward diagnostic tool for insomnia that efficiently assesses sleep status^49^. It comprises eight items that facilitate the diagnosis of insomnia. The eight multiple-choice items of the AIS can be easily adjusted to modify references to sleep induction, awakening during the night, final awakening, total sleep duration, sleep quality, well-being during the day, functioning capacity during the day, and sleepiness during the day. The scores for each item range from 0 (no problem) to 3 (very serious problem), with the total score ranging from 0 to 24. The total score cutoff for identifying pathological insomnia in AIS is six points^49^. The internal consistency coefficient of the AIS ranged from 0.78 to 0.88. The correlation between the AIS Japanese version and the Japanese versions of the Pittsburgh Sleep Quality Index and Insomnia Severity Index was 0.81 and 0.85, respectively^50,51^.

##### Severity of premenstrual syndrome (PMS)

The DRSP—developed as a symptom-recording tool for diagnosis^52^—is internationally recommended by the British Society of Obstetrics and Gynecology. Moreover, the DRSP is the most widely used PMS severity scale in clinical trials and comprises 24 items, 21 of which refer to PMS symptoms and three to issues that interfere with daily life—each of which is rated on a six-point scale ranging from 1 (not at all) to 6 (very severe).

During the development of the DRSP’s Japanese version, a shortened version was developed, with higher factorial validity than the Japanese version, being based on the classical test theory. It comprises eight symptom items. This has also been confirmed as a reliable and valid PMS scale^53^. When the total score of these 8 symptoms is 13 points or higher, the DRSP has a sensitivity of 90% and a specificity of 79% in identifying PMS-induced daily life disturbances (two points or higher on any of the three daily life disturbance items included in the DRSP)^54^. The eight-item short version of the J-DRSP showed good model fit in the confirmatory factor analysis (CFA) (Comparative Fit Index (CFI): 0.99, root mean square error of approximation (RMSEA): 0.048), and the intraclass correlation coefficients (ICC) values for the luteal and follicular phases were 0.61 (95% CI 0.51–0.68) and 0.70 (95% CI 0.62–0.77), respectively^54^.

##### Resilience

The 10-item connor-davidson resilience scale (CD-RISC 10)^55^ was used to evaluate psychological resilience. This is a 10-item, self-report scale developed to address certain limitations observed in the factor structure of the original 25-item Connor-davidson resilience scale (CD-RISC)^56^. The instrument uses a five-point Likert scale (from 0 = never to 4 = almost always). The final scores of the questionnaire were the total responses for each item, with a total possible score of 40. Higher scores indicate higher resilience levels. Reportedly, the CD-RISC 10 is a stable scale with excellent psychometric properties^55^. The reliability and validity of the Spanish version of the CD-RISC 10 were evaluated among young adults by Notario-Pacheco et al.^57^, who found that the scale exhibits satisfactory psychometric properties and a high degree of reliability (Cronbach’s α = 0.85), thus corroborating its single factor structure. Likewise, in this study, the reliability of the CD-RISC 10 was high (Cronbach’s α = 0.87). The reliability and validity of the Japanese version of CD-RISC 10 have not yet been clarified. However, the Chinese version of the CD-RISC 10 one-dimensional structure was supported by CFA. The fit indices indicated excellent model fit (CFI = 0.961, GFI = 0.972, RMSEA = 0.063, SRMR = 0.029). The standardized factor loadings ranged from 0.55 to 0.74 (all *p* < 0.001)^58^.

### Interviews on stabilizing the psychological state

Posters outlining the study’s purpose and requesting participation in a survey on the characteristics of female students at the IPU were placed on a bulletin board at the IPU. Independent interviews were conducted by members of the research team with extensive experience. The interviews were digitally recorded and lasted 30–60 min. All recordings were transcribed and anonymized. The observations (field notes) were recorded for each interview. Interviews continued until data saturation was reached, when new themes appeared infrequently, and code definitions were stable. All data were collected from November 9 to December 18, 2023. The transcripts were not returned to the participants for review.

Once data collection was complete, the data were analyzed iteratively using inductive thematic analysis. Two members of the team performed a targeted review of the transcripts and searched for discussions to create a code list. After reaching a consensus on the code list, each team member coded each transcript individually. Thereafter, three team members reviewed the reports for each code to identify overarching themes.

### Data analysis

SPSS 26.0 was used for statistical analysis. Data that did not follow a normal distribution were expressed as median and interquartile range (IQR). The Wilcoxon signed-rank test was used to compare the ME-FULLNESS app user and non-user groups. Results were considered statistically significant if *p* < 0.05.

### Contribution of the use of ME-FULLNESS, the psychological state following undergraduate and club activities, insomnia, PMS, and resilience

The app’s contribution to psychological state, insomnia, PMS, and resilience was evaluated using prediction one (sony network communications Inc. https://predictionone.sony.biz/). prediction one uses the open-source software neural network libraries (https://nnabla.org/) for deep learning. Based on the learning items, it automatically performs machine learning analysis such as neural networks and gradient boosting trees, and it is possible to easily and quickly create a prediction model using cross-validation^59^. The prediction model calculates the predicted value by entering the items for each factor. The details of the analysis are not disclosed. Using prediction one, it was estimated how much each factor of psychological state, insomnia, PMS and resilience, and the use of ME-FULLNESS, as well as undergraduate and club activities, contributed to the score indicating the psychological state (Fig. 5A).

## Results

### Response rate

Overall, 125 female students participated in the study (85 [68.0%] in the ME-FULLNESS user group and 40 [32.0%] in the non-user group). Of the participants, 101 (80.8%) completed the psychological test form after one month (76 (89.4%) in the ME-FULLNESS user group and 40 (62.5%) in the non-user group). Figure 3 presents the number of participants in each group.

### Stress and methods of improvement for female students

The female students reported that there was so much to learn in college that they sometimes had to quit club activities. Female students had several reasons for their difficulties in engaging in extracurricular activities. Some stated that they frequently have injuries or surgeries and need to recover quickly to participate in their target competitions and events. Others mentioned that occasionally, they were not selected as contestants because of their lack of abilities, such as being slow runners. They stated that to relieve stress, they praise themselves and run, buy, and eat all the desserts sold at convenience stores. Table 2 lists all representative quotes.

**Table 2:**
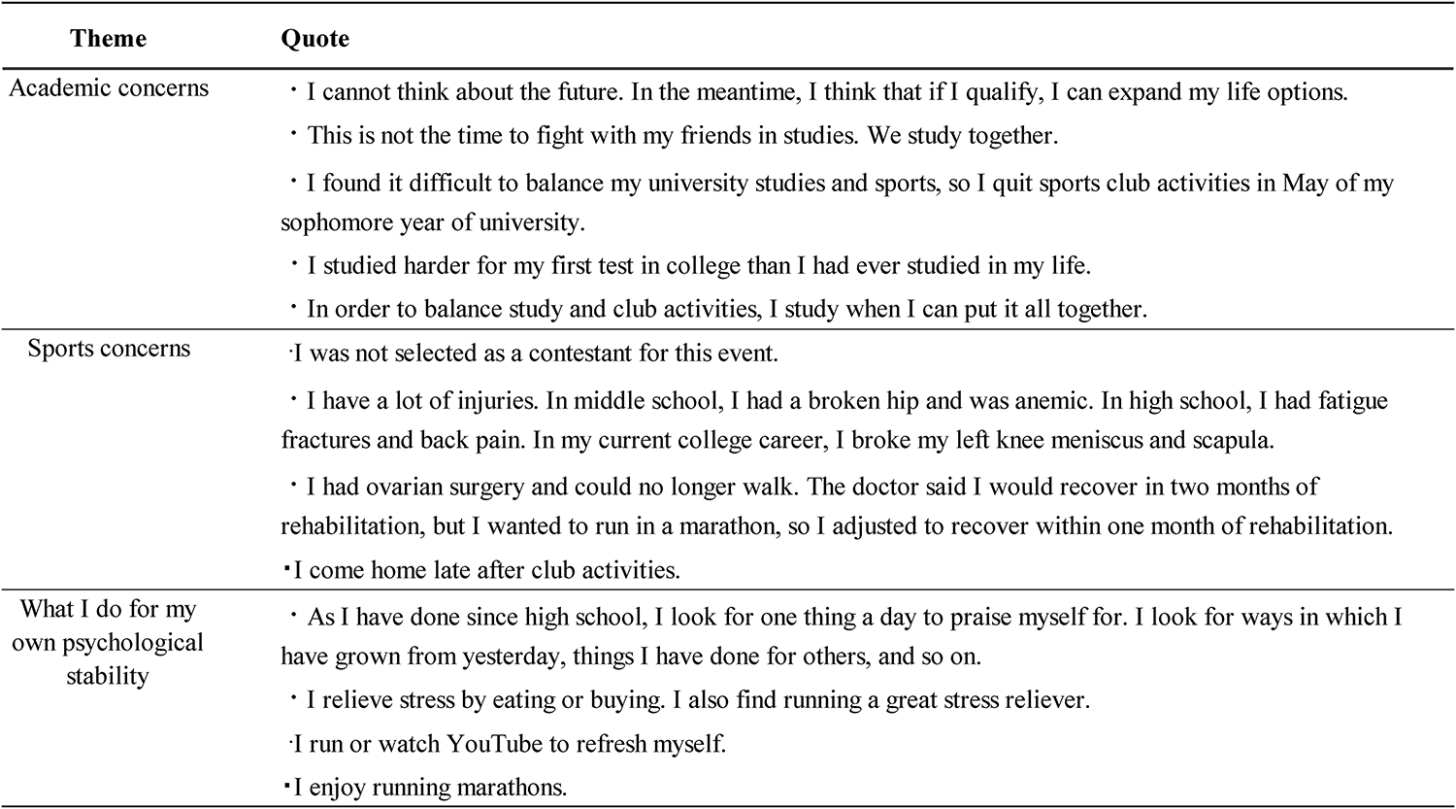
Representative statements from participants by key themes in academics and sports.

### Changes in the ME-FULLNESS application use

#### Percentage of psychological state in each group before and after using the ME-FULLNESS application

**Fig. 4:**
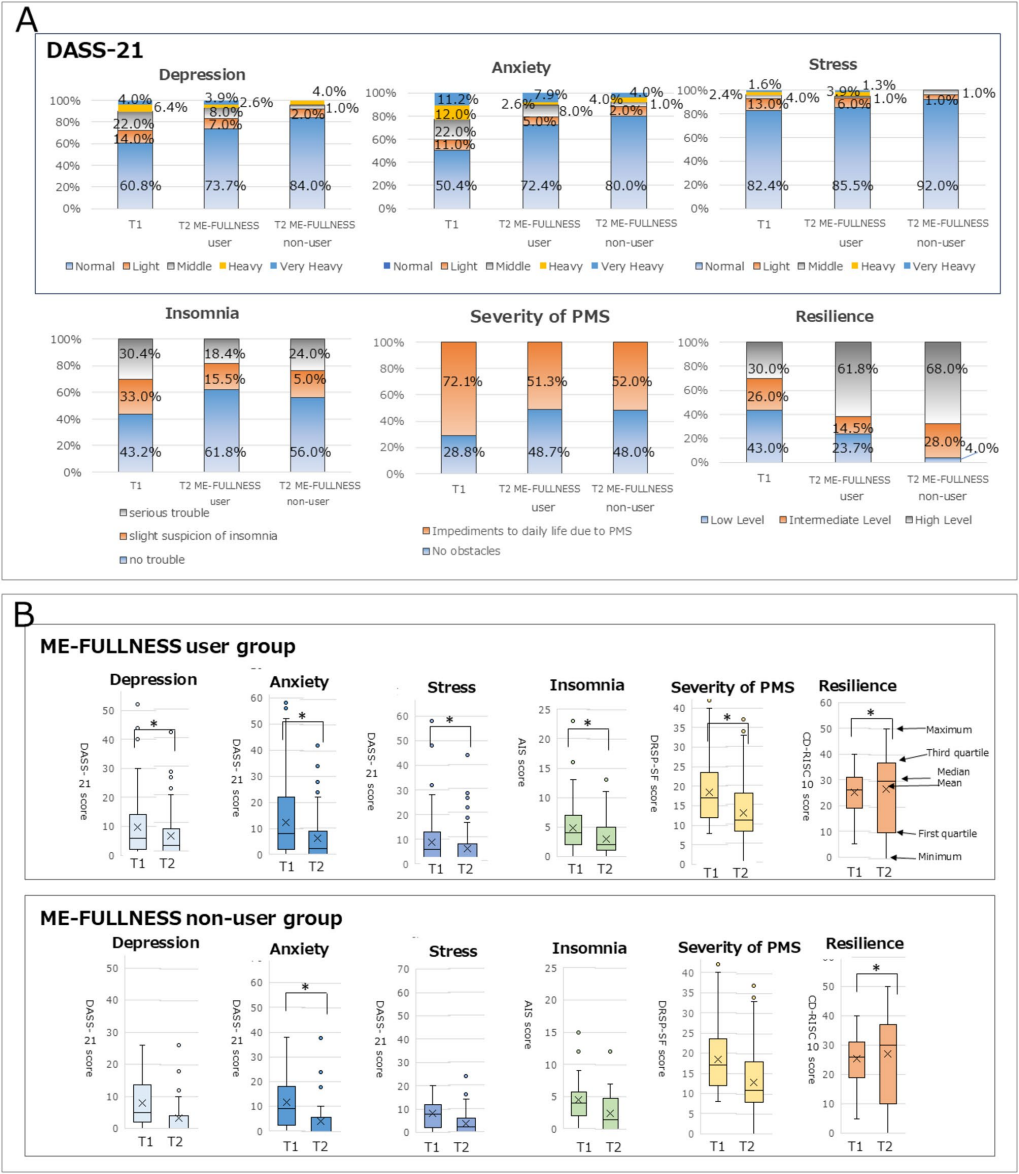
Changes brought about by using ME-FULLNESS. (**A**) Percentage of psychological state in each group before and after use of ME-FULLNESS^Ⓡ^ application; (**B**) Change in psychological test form scores for each group before and after using the ME-FULLNESS^Ⓡ^ application.

In this study, 101 subjects–—76 in the ME-FULLNESS application group, and 25 non-users (T2)—submitted psychological test forms (Fig. 4A).

#### Tests with correspondence before and after ME-FULLNESS use

The Wilcoxon signed-rank test results revealed that the ME-FULLNESS user group’s post-use DASS-21 scores were significantly lower than the pre-use DASS-21 scores (Depression *p* = 0.002; Anxiety *p* = 0.000; Stress *p* = 0.006). The ME-FULLNESS non-user group exhibited significantly lower post-use DASS-21 Anxiety scores than pre-use DASS-21 Anxiety scores (Depression *p* = 0.073; Anxiety *p* = 0.009; Stress *p* = 0.271) (Table 3). Figure 4B illustrates the ratio of the scores on the psychological questionnaire at T1 and T2.

**Table 3:**
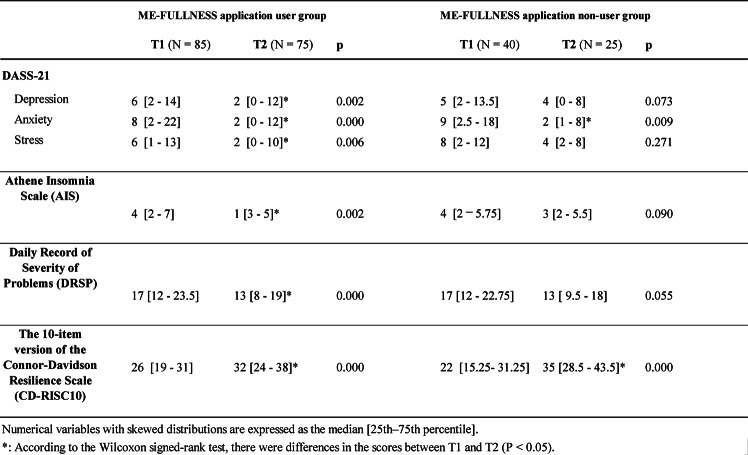
Representative statements from participants by key themes in academics and sports.

#### Analysis of psychological assessment before and after use of ME-FULLNESS by club activity

The ME-FULLNESS application use and non-use groups were divided by the club activity to which they belonged (Table 4), and the results of the analysis of psychological test form scores are presented. Comparisons of the respective T1 and T2 were made using the Wilcoxon rank-sum test method.

**Table 4:**
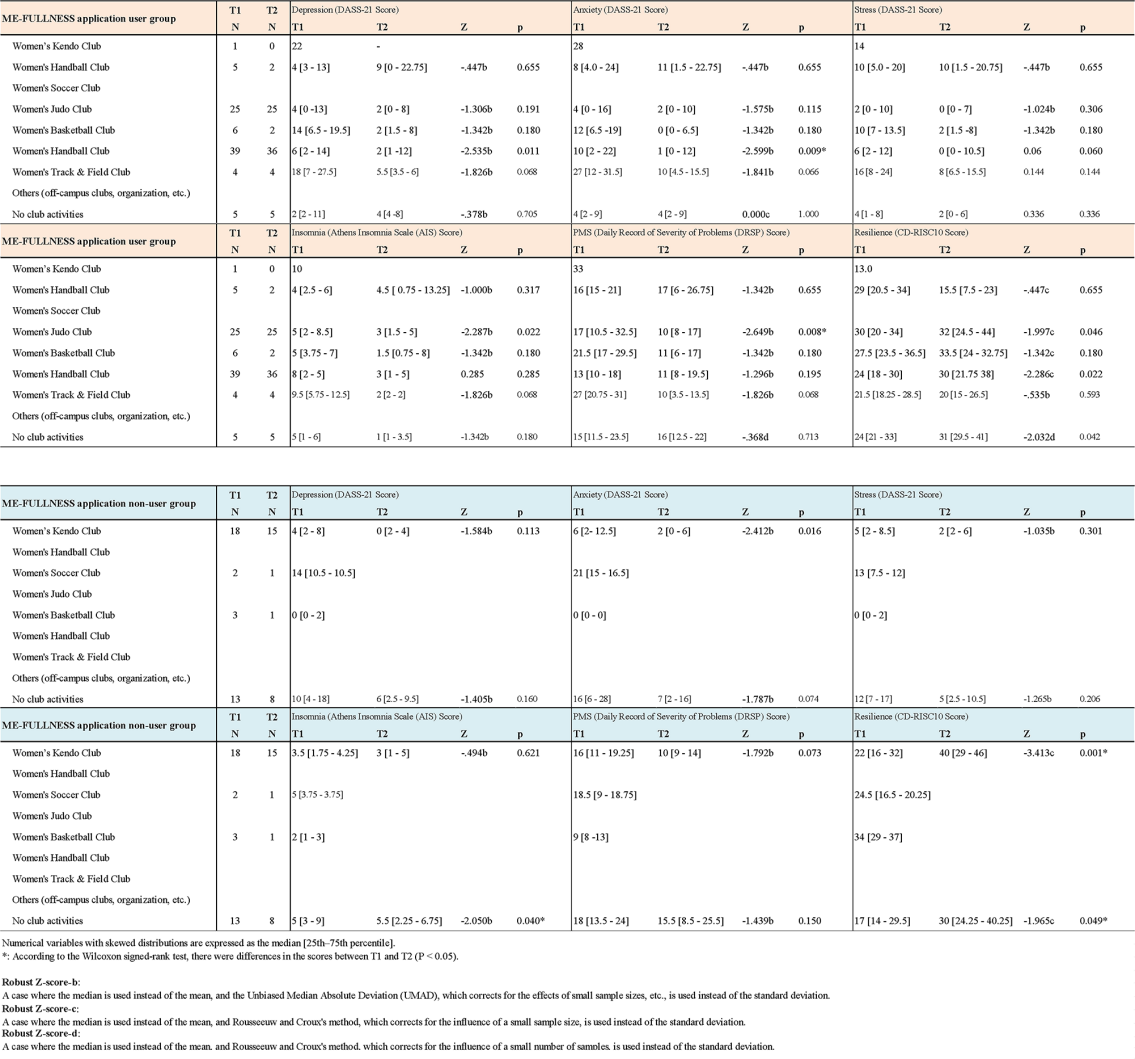
Change before and after the use of ML-FULLNESS for each club.

In the group using the ME-FULLNESS app, the median AIS score for the women’s judo team went from 5 [2–8.5: 25–75th percentile] to 3 [1.5–5: 25–75th percentile] (*p* = 0.022), and the DRSP score went from 17 [10.5–32.5: 25–75th percentile] to 10 [8–17: 25–75th percentile] (*p* = 0.008), a significant improvement. In the women’s handball group, the median DASS-21 depression score improved significantly from 6 [2–14: 25–75th percentile] to 2 [1–12: 25–75th percentile] and the median DASS-21 anxiety score improved from 10 [2–22: 25–75th percentile] to 1 [0–12: 25–75th percentile] (*p* = 0.009). The median CD-RISC10 score, indicative of resilience, increased significantly from 24 [18–30: 25–75th percentile] to 30 [21.75–38: 25–75th percentile] (*p* = 0.022). The median CD-RISC10 score significantly increased from 24 [21–35: 25–75th percentile] to 31 [29.5–33: 25–75th percentile] in the no club group (*p* = 0.042).

In the ME-FULLNESS non-user group, the median DASS-21 anxiety score significantly decreased from 6 [2–12.5: 25–75th percentile] to 2 [0–6: 25–75th percentile] in the female kendo group (*p* = 0.016) and the median CD-RISC10 score significantly increased from 22 [16–32: 25–75th percentile] to 40 [29–46: 25–75th percentile] (*p* = 0.001). The median AIS score significantly increased from 5 [3–9: 25–75th percentile] to 5.5 [2.25–6.75: 25–75th percentile] (*p* = 0.040) and the median CD-RISC10 score significantly increased from 17 [14–26.5: 25–75th percentile] to 30 [24.45–29.5: 25–75th percentile] (*p* = 0.049).

### Investigation of factors contributing to psychological state, insomnia, PMS, and resilience

**Fig. 5:**
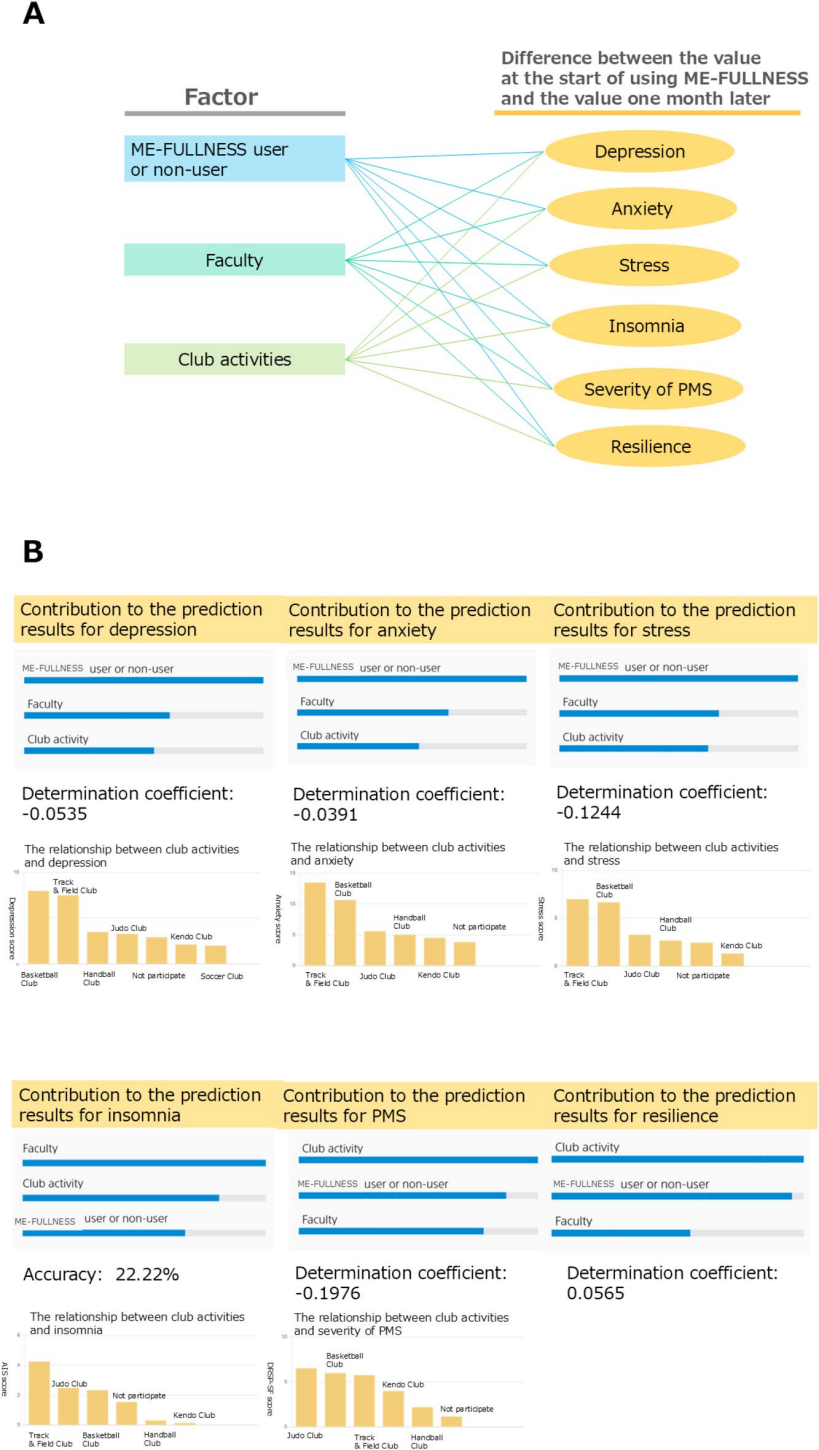
Examination of factors that contribute to psychological state, insomnia, PMS, and resilience. (**A**) Factors to be input into Prediction One and the target for obtaining the contribution rate; (**B**) Factors that contribute to psychological state, insomnia, PMS, and resilience using prediction one.

We used prediction one (Sony Communications Inc., Japan) to compare the contribution rates of ME-FULLNESS use, faculty, and club activities to psychological state, insomnia, PMS, and resilience (Fig. 5A). For all psychological states, ME-FULLNESS use was the most significant contributing factor. For insomnia, faculty was the most significant contributing factor. For PMS and resilience, club activities were the most significant contributing factors (Fig. 5B).

## Discussion

We carried out this study to determine the effect of smartphone apps on the management of mental health and PMS in female athletes. Smartphone apps are highly convenient and a promising intervention tool^60,61^. The psychological state, insomnia, PMS, and resilience of ME-FULLNESS users demonstrated a significant effect. However, non-users of ME-FULLNESS also showed a significant change in anxiety and resilience.

One possible reason for this is that participation in the survey may have played a more important role in forming intrinsic motivation than the existence of the app for anxiety and resilience^62^. Intrinsic motivation is strongly linked to self-efficacy, and for students, this means that they may have been able to experience meaningful improvements in anxiety and resilience regardless of whether they used the app or not^63,64^.

The results of this study indicate that support from digital devices can contribute to the psychological well-being of female students at a physical education university, regardless of their faculty or the type of club activities they participate in. Furthermore, support for insomnia may be provided in the following order: teachers, club activities, and digital devices. Regarding PMS and resilience, it is thought that digital device support has the greatest impact, followed by the type of club activities and teachers. These results are consistent with those of previous studies on the ME-FULLNESS app, supporting the stability of its effects. However, because this research design was not randomized, it is difficult to accurately confirm the effects of ME-FULLNESS.

We tried to produce trends for each type of club activity; however, the number of people belonging to each club activity was uneven and there were also club activities that were unsuitable for statistical processing. To examine the uniqueness of each club, it was necessary to increase the number of people belonging to each club. To solve this problem, we decided to use Prediction One^59^, which creates predictive models for the ME-FULLNESS app, faculties, and clubs using machine learning even with a small number of people, to determine the contribution to psychological state, insomnia, PMS severity, and resilience (Fig. 5A, B).

Additionally, we surveyed 35 town employees who used the application for one month in Shichigahama, a town affected by the 2011 Great East Japan Earthquake; the results indicated that the percentage of those indicating insomnia and percentage of those indicating stress decreased^65^. The relaxation effects derived from the application used in this study are supported by Motokawa et al.’s finding that the ME-FULLNESS application improves subjective stress markers and salivary cortisol levels^34^. The ME-FULLNESS application introduces the concept of “the experience of relaxation through slow rhythmic changes”^34^. Prior research supports the idea that slow rhythmic changes induce relaxation, as observed in slow breathing^66–71^, music with a decreasing tempo^72^, and gentle rocking movements^73^. Utilizing these findings, the ME-FULLNESS application is an easy-to-use stress care system that can be integrated into daily life.

Several previous studies have noted the improvement in insomnia based on the ME-FULLNESS app. Peak Sleep is a smartphone app based on scientifically validated principles for improving sleep quality, such as mindfulness meditation and cognitive behavioral therapy^74^. However, for Peak Sleep, no difference has been noted between app-using and non-app-using groups. Additionally, digital CBT-I (somnio, mementor DE GmbH, Leipzig, Germany) is reportedly effective for sleep, but no differences in effectiveness have been reported between users and non-users for anxiety and depression symptoms, physical health, and quality of life^75^.

Further, Flo—an app focused on menstrual and reproductive health^76^—is designed to enhance menstrual and reproductive health literacy and has demonstrated improvements in menstrual health knowledge and awareness, general health and well-being, and PMS or premenstrual dysphoric disorder (PMDD) symptom burden after three months of use. Although the ME-FULLNESS application did not specifically provide menstrual or reproductive information, PMS symptoms improved in the group that used the application, suggesting that relaxation during the application may have improved PMS symptoms.

Both the app-using and non-app-using groups exhibited significant improvements in resilience. That is, the improvement in resilience was not considered an effect of app use. Song et al.^77^ found that self-esteem was the most influential factor in the development and improvement of psychological resilience in a study of college students. The participation of IPU students in our study may have helped their self-esteem without the use of a fullness application.

## Limitations

Several limitations should be noted in the study design of the ME-FULLNESS app for female athletes in university or college. This intervention study included the use of a non-randomized design due to practical constraints^78^. According to the table in the paper by Schichido^57^ and Lui et al.^58^, the number of samples required for a repeated measurement design for a continuous variable is 64. However, it is also possible that the results of studies obtained through non-randomized sampling differ from those obtained through random sampling. A methodological index for non-randomized studies (MINORS) is an effective tool designed to assess the methodological quality of non-randomized surgical studies, regardless of whether they are comparative or non-comparative^79^; however, this tool was not adopted in this study.

Another limitation of this study was the difference in the number of participants in the ME-FULLNESS user and non-user groups. As the participants were female students at a physical education university, it would be unethical to include participants who wished to use ME-FULLNESS in the non-user group. When comparing the differences between the ME-FULLNESS user and non-user groups, the small number of participants in the non-user group may have had an impact. An additional limitation was the lack of initial assessment of the ME-FULLNESS use and non-use groups before the start of the study.

Another limitation of this study involved limited generalizability, as the participants were only students at a certain physical education university. Therefore, the results may not be representative of female athletes at all physical education universities in Japan, and the effectiveness of this application should be verified at other universities as well. Furthermore, all psychological assessments used were self-assessed. Self-reports are subjective and can be influenced by social desirability bias or participants’ mood at the time of assessment^80^.

In addition, this study lacked long-term follow-up. This study only used the ME-FULLNESS application for one month, and the intervention period may have been too short. However, in a study of digital psychological interventions, the JoyPop™ smartphone app^81^ for university students^82^ and the Understanding Mindfulness App^83^ had intervention periods of 28 days; thus, this intervention period may have been appropriate. Moreover, the frequency and duration of use were left to the discretion of the ME-FULLNESS user; thus, the best time and frequency of use have not yet been determined. A study on the myPlan app for the health and safety of female university students who experienced violence from a partner investigated its long-term effects (6 and 12 months after the start of the survey), and demonstrated the app’s effectiveness after 12 months^84^. Similarly, in this study, it would be worth investigating the app’s effects not only one month after the intervention but also 6 and 12 months later.

We attempted to produce trends by type of club activity, but the number of students belonging to each club activity was mixed, and some club activities had only a small number of students who were not suitable for statistical processing. To examine the specificity of each club activity, it is necessary to increase the number of people who belong to one type of club activity. Therefore, future studies should recruit a wider range of participants.

Finally, high-quality survey estimates and the minimization of bias are necessary for the representativeness of the survey results for the target population. By improving the participation rate of the survey, external validity is increased, respondent bias is reduced, and more representative estimates are obtained^85^. In this study, the participation rate after using ME-FULLNESS was 89% for the users. However, the participation rate for non-users of ME-FULLNESS was 63%, and there is a possibility of respondent bias.

## Conclusion

The results of this study show that using the ME-FULLNESS app for female students at a physical education university may contribute significantly to improving sports performance by improving psychological states, contributing to the potential of digital interventions in sports. However, the effect size of the ME-FULLNESS app is significant but modest for insomnia, severity of PMS, and resilience. To strengthen the effect model of the ME-FULLNESS app, it is necessary to adjust for potential confounding variables. It appears that the department to which one belongs has a stronger effect on insomnia than the ME-FULLNESS app, and the type of club one is in has a stronger effect on the severity of PMS and resilience than the ME-FULLNESS app. Therefore, factors such as the department and type of club should be carefully considered when developing and implementing new digital interventions for female students at physical education universities. It would be feasible, affordable, and effective to ensure that the digital intervention has a lasting impact on the overall well-being of female students who are required to achieve good academic results and sports performance at university.

The ME-FULLNESS app is thus a resource that can be accessed 24 h a day, 365 days a year via a smartphone, and it promotes the well-being of female students at a physical education university. While this research was conducted at one private physical education university in Japan, IPU, and the athletes affiliated with IPU’s athletic clubs performed well in international sports competitions, it is thought that using the ME-FULLNESS app as an intervention is also promising for female students at other physical education universities who have achieved results at a global level.

## Data Availability

The datasets generated and/or analysed during the current study are not publicly available due to privacy and ethical restrictions but are available from the corresponding author on reasonable request.
